# Mg_12_O_12_ and Be_12_O_12_ Nanocages as Sorbents and Sensors for H_2_S and SO_2_ Gases: A Theoretical Approach

**DOI:** 10.3390/nano12101757

**Published:** 2022-05-21

**Authors:** H. M. Badran, Kh. M. Eid, Sotirios Baskoutas, H. Y. Ammar

**Affiliations:** 1 Physics Department, College of Science and Arts, Najran University, Najran 11001, Saudi Arabia; hmbadran@nu.edu.sa; 2 Physics Department, Faculty of Education, Ain Shams University, Cairo 11566, Egypt; khmeid@yahoo.com; 3 Department of Physics, College of Science and Arts, Qassim University, Albukayriyah 52725, Saudi Arabia; 4 Department of Materials Science, University of Patras, 26504 Patras, Greece; bask@upatras.gr

**Keywords:** adsorption of H_2_S and SO_2_, BeO and MgO nano-cages, density functional theory, thermodynamics, molecular dynamics

## Abstract

Theoretical calculations based on the Density Functional Theory (DFT) have been performed to investigate the interaction of H_2_S as well SO_2_ gaseous molecules at the surfaces of Be_12_O_12_ and Mg_12_O_12_ nano-cages. The results show that a Mg_12_O_12_ nano-cage is a better sorbent than a Be_12_O_12_ nano-cage for the considered gases. Moreover, the ability of SO_2_ gas to be adsorbed is higher than that of H_2_S gas. The HOMO–LUMO gap (E_g_) of Be_12_O_12_ nano-cage is more sensitive to SO_2_ than H_2_S adsorption, while the E_g_ value of Mg_12_O_12_ nano-cage reveals higher sensitivity to H_2_S than SO_2_ adsorption. The molecular dynamic calculations show that the H_2_S molecule cannot be retained at the surface of a Be_12_O_12_ nano-cage within 300–700 K and cannot be retained on a Mg_12_O_12_ nano-cage at 700 K, while the SO_2_ molecule can be retained at the surfaces of Be_12_O_12_ and Mg_12_O_12_ nano-cages up to 700 K. Moreover, the thermodynamic calculations indicate that the reactions between H_2_S as well SO_2_ with Be_12_O_12_ and Mg_12_O_12_ nano-cages are exothermic. Our results suggest that we can use Be_12_O_12_ and Mg_12_O_12_ nano-cages as sorbents as well as sensors for H_2_S and SO_2_ gases.

## 1. Introduction

Recently, great efforts have been made to develop novel gas sensors and detectors as well as gas-removing materials. This is to control the pollutant gases broadly produced from industrial activities and burning fuel, etc. The toxic H_2_S and SO_2_ gases are produced as byproducts from SF6 decomposition, which is widely used as an insulating gas in high-voltage transformers and circuit breakers [1,2,3,4]. H_2_S is mostly found in crude petroleum, natural gas, and coal gasification. In addition, some organic materials decompose, releasing H_2_S [5,6,7]. H_2_S is also released in many industries such as the paper industry and biomass fermenters [7,8]. The combustion of sulfur-containing fossil fuels releases SO_2_ into the air, and SO_2_ is naturally released as a byproduct of volcanic activity [9,10]. The H_2_S as well SO_2_ gases pose several hazards to the environment and human health. H_2_S exposure leads to coughing, eye irritation, and a runny nose, harms the nervous system by killing the neurons and may cause death [11,12]. Moreover, H_2_S is a corrosive gas and has devastating impacts on industrial catalysts [7,8]. SO_2_ interacts with the air resulting in acidic rain which causes the corrosion of metals and disintegration of buildings [9,10]. Furthermore, SO_2_ causes skin burning, eye irritation and respiratory system inflammation, and may cause death [7,13,14,15]. Therefore, several attempts have been made to utilize many materials as sorbents and detectors for H_2_S and SO_2_ gases, such as fullerene-like gallium nitride [16], CuO(111) surface [17], pristine graphene and graphene oxide [18], NH-decorated graphene [8], activated carbon, [19,20], pillared clays [21], zeolites [22], p-CuO/n-ZnO Heterojunction [23], Cu (100) and Au (100) surfaces [24,25], Cu doped MoSe_2_ [12], monolayer Janus MoSSe [26], aza-macrocycle [27], Cu-modified and Cu-embedded WSe_2_ monolayers [28], porous N_4_O_4_-donor macrocyclic Schiff base [29] and MgO (100) surfaces [10].

Metal oxides have a significant consideration due to their several applications. They are usually utilized as substrates for epitaxial growth of multilayers and clusters, catalytic processes, hydrogen storage materials, sensors and sorbent materials [10,30,31,32,33,34]. Recently, nano-structures have been frequently utilized as gas detectors and sorbents due to their appropriate features such as their tiny size, precision, and reactivity [35,36,37].

Furthermore, Shamlouei et al. reported that nanocages with a X_12_Y_12_ structure are the most stable among (XY)_n_ nano-cages [38]. Ren et al. [39] have investigated (BeO)_N_ clusters and found that Be_12_O_12_ is one of the most feasible nano-cages. Ziemann and Castleman [40] have proven that a Mg_12_O_12_ nano-cage displays unique stability among (MgO)_n_, n ≤ 90 nano-structures. The Mg-O bond in Mg_12_O_12_ nano-cages has an ionic character [38], while the Be-O bond has ionic and covalent characters [41], therefore one can expect different applications for Be_12_O_12_ and Mg_12_O_12_ nano-cages. Be_12_O_12_ and Mg_12_O_12_ have several applications; for instance, Be_12_O_12_ has been utilized as a catalyst to convert CH_4_ into CH_3_OH [42], an electro-conductive sensor for sulfur mustard [43], and a detector and sorbent for Mercaptopyridine [44], and Be_12_O_12_ and Mg_12_O_12_ have been utilized for hydrogen storage applications [45,46], detection and adsorption of Tabun [47]

According to our knowledge, the interaction of H_2_S and SO_2_ gases at the surfaces of Be_12_O_12_ and Mg_12_O_12_ nano-cages has not been investigated yet. Hence, the present work aims to shed light on the characteristics of the interaction of H_2_S and SO_2_ with Be_12_O_12_ and Mg_12_O_12_ nano-cages for adsorption and sensing applications using DFT calculations.

## 2. Methods

To investigate the adsorption characteristics of H_2_S and SO_2_ molecules onto Be_12_O_12_ and Mg_12_O_12_ nano-cages, DFT and DFT-D3 methods [48] are used at the B3LYP/6-31G(d,p) level. D3 is a version of Grimme’s dispersion [49]. B3 is Becke’s three-parameter exchange functional [50] and LYP is the correlation functional of Lee, Yang and Parr [51,52]. A geometrical optimization without any restriction is performed for the free gaseous molecules, bare nano-cages and gas/nano-cage complexes. The ionization potential (IP) is calculated as [10,37]:

(1) $$\mathrm{IP}=E_{\mathrm{nano}-{\mathrm{cage}}^{+}\;}-E_{\mathrm{nano}-\mathrm{cage}\;}$$

where $E_{\mathrm{nano}-{\mathrm{cage}}^{+}\;}$ is the energy of the nano-cage with one electron lost at the same geometrical structure of the neutrally charged nano-cage. The chemical potential (µ), hardness (η) and electrophilicity (ω) are calculated as [53,54]:

(2) $$\mu \approx -\frac{1}{2}\;\left(E_{\mathrm{HOMO}}+E_{\mathrm{LUMO}}\right)$$

(3) $$\eta \approx \frac{1}{2}\;\left(E_{\mathrm{LUMO}}-E_{\mathrm{HOMO}}\right)$$

(4) $$\omega \approx \frac{\mu^{2}}{2\eta }$$

Molecular dynamic simulations via the Atom Centered Density Matrix Propagation molecular dynamics model (ADMP) as implemented in Gaussian 09 package are achieved for the investigated nano-cages and gas/nano-cage complexes.

The adsorption energy (E_ads_) and the corrected adsorption energy ($E_{\mathrm{ads}}^{\mathrm{corr}}$) with basis set superposition error (BSSE) have been estimated as [55]:

(5) $$E_{\mathrm{ads}}=E_{\mathrm{gas}/\mathrm{nano}-\mathrm{cage}}-\left(E_{\mathrm{gas}}+E_{\mathrm{nano}-\mathrm{cage}}\right)$$

(6) $$E_{\mathrm{ads}}^{\mathrm{corr}}=E_{\mathrm{ads}}+E_{\mathrm{BSSE}}$$

where ${\;E}_{\mathrm{gas}/\mathrm{nano}-\mathrm{cage}}$, $E_{\mathrm{gas}}$, and $E_{\mathrm{nano}-\mathrm{cage}}$ are the energies of gas/nano-cage complexes, free gas molecules, and bare nano-cages, respectively. The charge density difference (Δρ) for the complexes is computed as:

(7) $$\Delta \rho =\rho_{\mathrm{gas}/\mathrm{nano}-\mathrm{cage}}-\left(\rho_{\mathrm{gas}}+\rho_{\mathrm{nano}-\mathrm{cage}}\right)$$

where $\;\rho_{\mathrm{gas}/\mathrm{nano}-\mathrm{cage}}$, $\rho_{\mathrm{gas}}$, and $\rho_{\mathrm{nano}-\mathrm{cage}}$ are the charge densities for gas/nano-cage complexes, free gas molecules, and bare nano-cages, respectively.

Thermodynamic calculations are performed via vibrational calculations to predict enthalpies as well free energies for the considered gases, nano-cages, and gas-cages complexes. Enthalpy difference (ΔH) and free energy difference (ΔG) for gas/nano-cage complexes are evaluated as [56]:

(8) $$\Delta H=H_{\mathrm{gas}/\mathrm{nano}-\mathrm{cage}}-\left(H_{\mathrm{gas}}+H_{\mathrm{nano}-\mathrm{cage}}\right)$$

where $H_{\mathrm{gas}/\mathrm{nano}-\mathrm{cage}}$, $H_{\mathrm{nano}-\mathrm{cage}}$, and $H_{\mathrm{gas}}$ are the enthalpies for gas/nano-cage complexes, bare nano-cages and free gas molecules, respectively.

(9) $$\Delta G=G_{\mathrm{gas}/\mathrm{nano}-\mathrm{cage}}-\left(G_{\mathrm{gas}}+G_{\mathrm{nano}-\mathrm{cage}}\right)$$

where $G_{\mathrm{gas}/\mathrm{nano}-\mathrm{cage}}$, $G_{\mathrm{nano}-\mathrm{cage}}$, and $G_{\mathrm{gas}}$ are the free energies for gas/nano-cage complexes, bare nano-cages_,_ and free gas molecules, respectively.

All the calculations have been carried out by Gaussian 09 program package [57]. GaussSum3.0 program is used to visualize the densities of states (DOS) [58]. Atomic charges are calculated for the considered structures via full natural bond orbital (NBO) analyses by using NBO version 3.1 [59].

## 3. Results and Discussions

### 3.1. Structural and Electronic Properties of Be_12_O_12_ and Mg_12_O_12_

**Figure 1:**
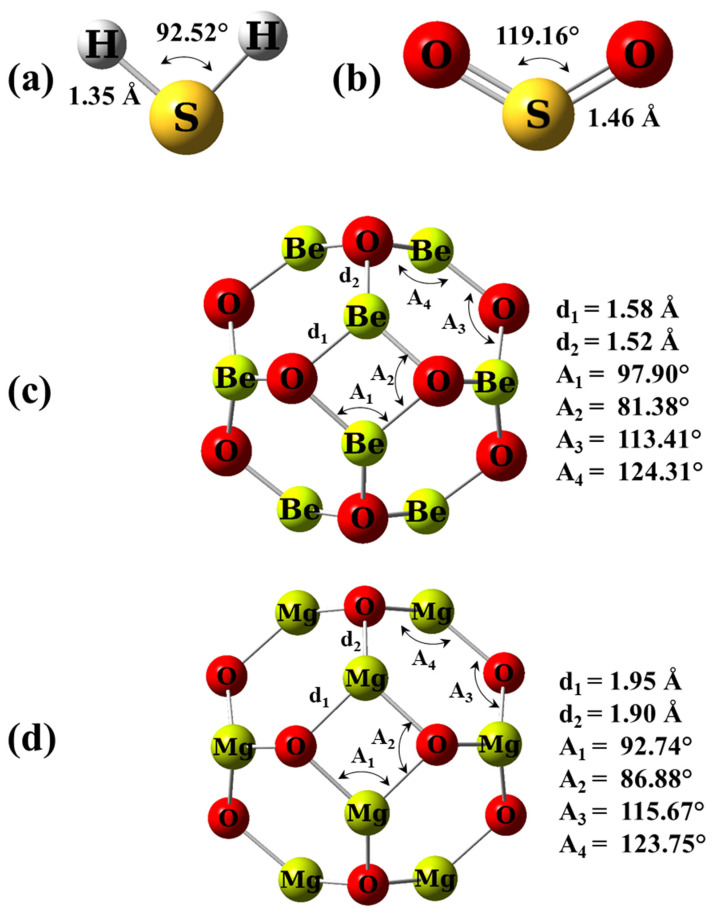
The optimized structures for (**a**) free H_2_S gas, (**b**) free SO_2_ gas, (**c**) Be_12_O_12_, and (**d**) Mg_12_O_12_ nano-cages.

The optimized structures for the scrutinized adsorbed gases H_2_S and SO_2_, as well the adsorbent nano-cages Be_12_O_12_ and Mg_12_O_12_, are shown in Figure 1.

For H_2_S gas, the S-H bond length and the H-S-H angle are 1.35 Å and 92.52°, respectively, while for SO_2_ gas, the S-O bond length and the O-S-O angle are 1.46 Å and 119.16°, respectively, in good agreement with previous studies [10,29]. Be_12_O_12_, as well Mg_12_O_12_ nano-cages are constructed of eight hexagonal and six tetragonal rings. It is noticed that all the metallic (Be and Mg) and O sites are identical. These nano-cages have two metal–oxygen bond types. They are denoted as d_1_ and d_2_ in Figure 1, where d_1_ shares a hexagon ring and a tetragon ring while d_2_ shares two hexagon rings. The d_1_ values are 1.58 and 1.95 Å, whereas the d_2_ values are 1.52 and 1.90 Å for Be_12_O_12_ and Mg_12_O_12_, respectively, match well with the previous studies [39,60,61,62,63]. Table 1 represents the electronic properties of Be_12_O_12_ and Mg_12_O_12_ nano-cages. 

**Figure 2:**
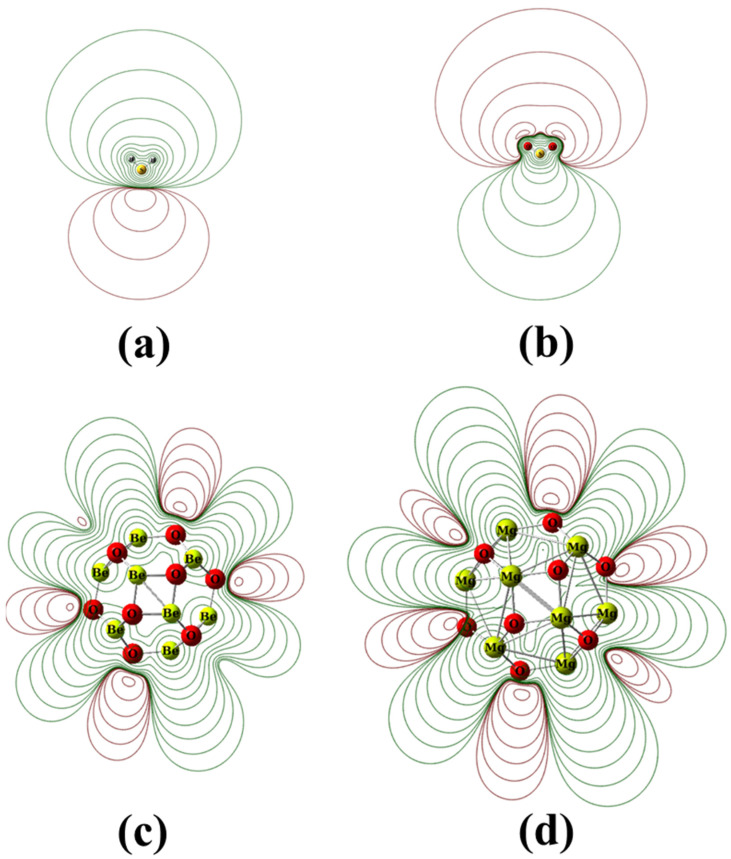
The molecular electrostatic potential contours (MESP) for (**a**) free H_2_S gas, (**b**) free SO_2_ gas, (**c**) Be_12_O_12_, and (**d**) Mg_12_O_12_ nano-cages at ±0.001, ±0.002, ±0.004,…, ±0.8 au iso-values. Red and green colors are assigned to negative and positive values, respectively.

The HOMO–LUMO energy gap (E_g_) values for Be_12_O_12_ and Mg_12_O_12_ are 7.829 and 4.839 eV while the ionization potential (IP) values are 10.273 and 7.983 eV, respectively, in good agreement with the previous studies [10,30,31,39,44,60,61,62,63]. The lower IP value for the Mg_12_O_12_ nano-cage suggests its higher ability to donate electrons than the Be_12_O_12_ nano-cage. The atomic charges for the investigated nano-cage are calculated via natural bond orbital (NBO) analysis. For Be_12_O_12_, the atomic charges for Be and O sites are 1.186 and −1.186 e, while for Mg_12_O_12_, the atomic charges for Mg and O sites are 1.442 and −1.442 e, respectively. In other words, the charge polarization for the Mg-O bond is greater than that for the Be-O bond; therefore, the Mg_12_O_12_ is expected to be more reactive than Be_12_O_12_. This matches the calculated E_g_, IP, η, and ω values, where the higher chemical stability and consequently lower reactivity for a molecule are marked by wide E_g_, large IP, η, and low ω values [64,65,66,67]. Figure 2 illustrates the molecular electrostatic potential (MESP) for H_2_S, SO_2_, Be_12_O_12_, and Mg_12_O_12_. 

It is clear that the S atom is surrounded by negative and positive electrostatic potentials for H_2_S and SO_2_ molecules, respectively. Furthermore, the MESP around the SO_2_ molecule is extended in space more than that of the H_2_S molecule.

For Be_12_O_12_ and Mg_12_O_12_ nano-cages, the O atoms and the metallic atoms are surrounded by negative and positive electrostatic potentials, respectively. In addition, the MESP of Mg_12_O_12_ is more extended around the nano-cage than that of the Be_12_O_12_ nano-cage. This is due to the higher charge polarization of Mg_12_O_12_. Therefore, it is expected that the S atoms of H_2_S and SO_2_ tend to be attracted to the metallic sites and oxygen sites of the nano-cages, respectively. Furthermore, the electric dipole moment (D) of Be_12_O_12_ and Mg_12_O_12_ nano-cages are 0.001 and 0.010 Debye, respectively. The low D values are owing to the uniform charge distribution on the nano-cages.

**Figure 3:**
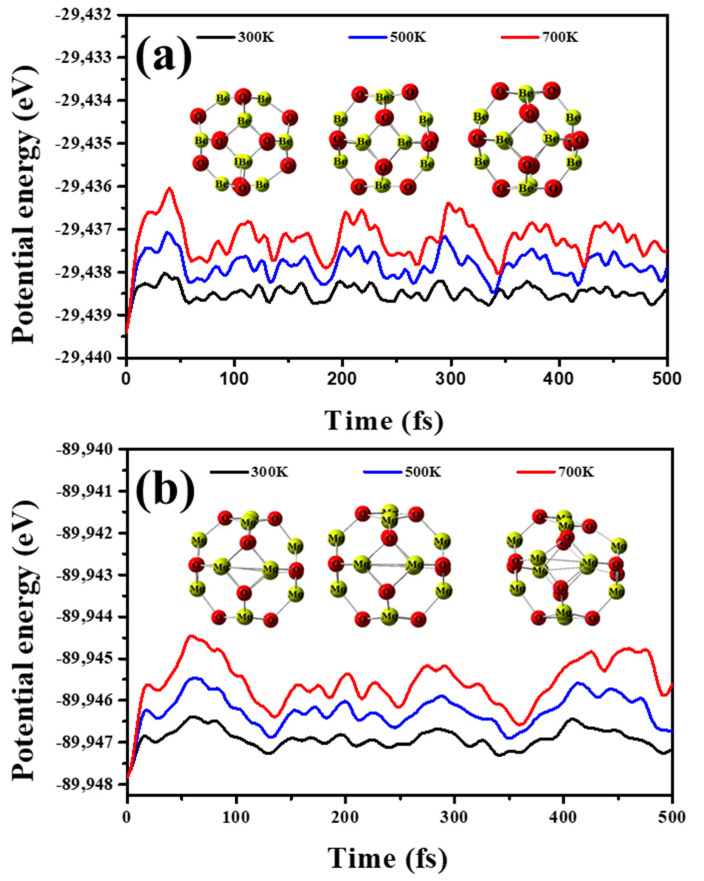
Potential energy fluctuations during MD simulation as well as the atomic configuration after 500 fs at 300, 500, and 700 K for (**a**) Be_12_O_12_, and (**b**) Mg_12_O_12_ nano-cages.

Molecular dynamic (MD) simulations examine the stability of the considered nano-cages at 300, 500, 700 K for a total time of 500 fs. Figure 3 depicts the fluctuation of the potential energy versus the time and the nano-cage geometric configuration at the end of the period.

It is obvious the potential energy trivially varies and no considerable distortion is observed for the nano-cages; this emphasizes the stability of Be_12_O_12_ and Mg_12_O_12_. In addition, the optimized geometries of discussed nano-cages were verified as true minima on the potential energy surfaces by the absence of imaginary frequencies [68,69,70,71].

### 3.2. Adsorption of H_2_S and SO_2_ Gases

**Figure 4:**
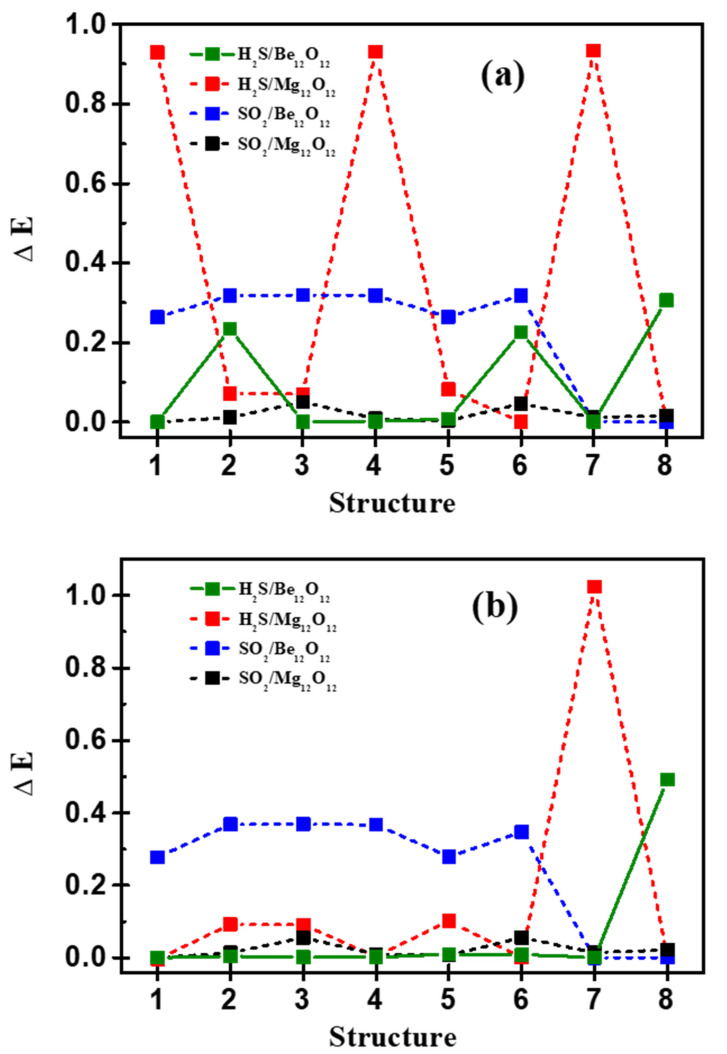
The differences in total electronic energies (ΔE) for the examined structures calculated at (**a**) DFT and (**b**) DFT-D3.

DFT as well DFT-D3 calculations were performed to investigate the adsorption characteristics of the adsorbed gases H_2_S and SO_2_ at the surfaces of Be_12_O_12_, as well Mg_12_O_12_, nano-cages. Four complexes are investigated—H_2_S/Be_12_O_12_, H_2_S/Mg_12_O_12_, SO_2_/Be_12_O_12_, and SO_2_/Mg_12_O_12_. There are several possibilities of the gas interaction with the nano-cage, therefore eight adsorption modes for each complex have been fully optimized without any restrictions. Tables S1 and S2 in the Supplementary Data show the examined adsorption modes for H_2_S interaction with Be_12_O_12_ and Mg_12_O_12_, respectively. We found that the H_2_S prefers to interact via its S atom toward the metallic atom Be or Mg of the nano-cage. Tables S3 and S4 in the Supplementary Data show the examined adsorption modes for SO_2_ interaction with Be_12_O_12_ and Mg_12_O_12_, respectively. One can observe that SO_2_ prefers to interact via its S and O atoms toward the O sites and the metallic atoms, respectively, of the nano-cages. The differences in total electronic energies (ΔE) for the examined orientations are shown in Figure 4.

**Figure 5:**
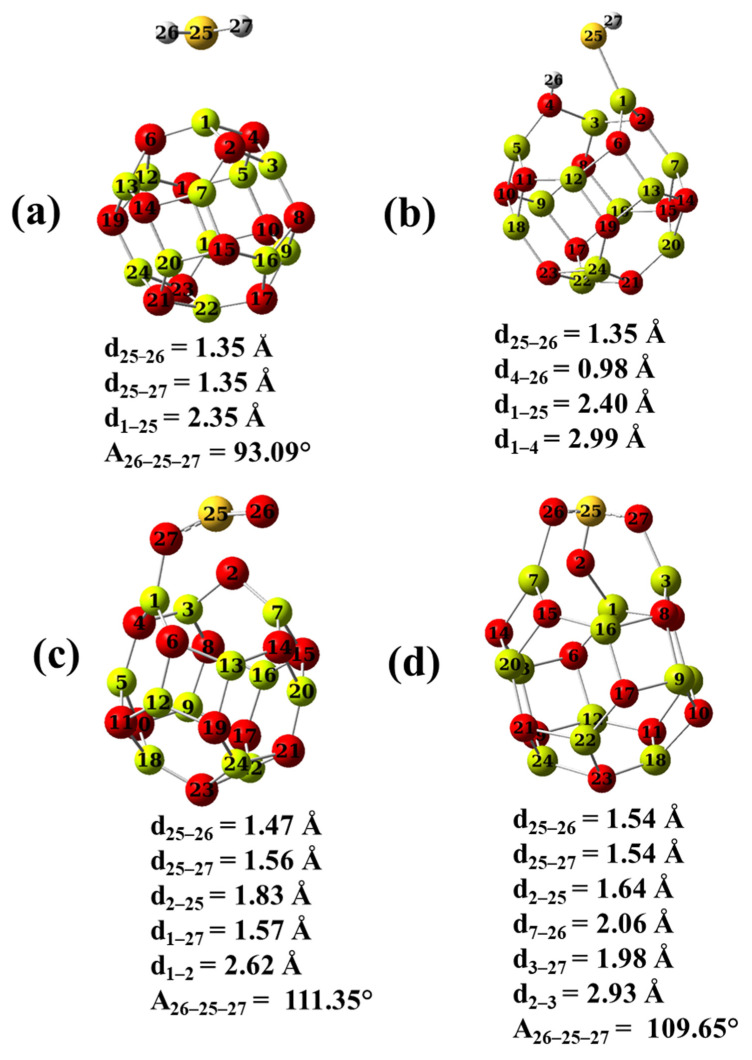
The optimal structures for (**a**) H_2_S/Be_12_O_12_, (**b**) H_2_S/Mg_12_O_12_, (**c**) SO_2_/Be_12_O_12_, and (**d**) SO_2_/Mg_12_O_12_ complexes.

DFT and DFT-D3 calculations show that modes 1, 6, 8, and 1 for H_2_S/Be_12_O_12_, H_2_S/Mg_12_O_12_, SO_2_/Be_12_O_12_, and SO_2_/Mg_12_O_12_, respectively, are the most energetically stable adsorption modes. Figure 5 presents the most energetically stable adsorption modes that have more negative adsorption energy for each complex.

Table 2 list the adsorption properties of H_2_S and SO_2_. Notably, the DFT-D3 calculations give more negative adsorption energy values than DFT calculations. 

In contrast, the values for HOMO, LUMO, E_g_, Q, and D have no considerable variations between DFT and DFT-D3 calculations. The $E_{\mathrm{ads}}^{\mathrm{corr}}$ values show that the interaction of H_2_S as well SO_2_ at the surfaces of Be_12_O_12_ and Mg_12_O_12_ is a chemical interaction, where the $E_{\mathrm{ads}}^{\mathrm{corr}}$ values are lower than −0.2 eV [67]. The released adsorption energy is in the following trend SO_2_/Mg_12_O_12_ > H_2_S/Mg_12_O_12_ > SO_2_/Be_12_O_12_ > H_2_S/Be_12_O_12_. One can notice that the ability of the Mg_12_O_12_ nano-cage to adsorb the considered gases is higher than that of the Be_12_O_12_ nano-cage. This is referred to as the higher ability of the Mg_12_O_12_ nano-cage to donate electrons than the Be_12_O_12_ nano-cage. Additionally, the ability of the SO_2_ gas to be adsorbed on the Mg_12_O_12_ nano-cage, as well as the Be_12_O_12_ nano-cage, is higher than that of H_2_S gas. Figure 5a shows that the H_2_S/Be_12_O_12_ complex, the S atom of the H_2_S molecule, is bonded to a Be site at a distance (d_1–25_) of 2.35 Å, the S-H bond length is the same as that of the free H_2_S molecule, and there is a negligible increase of the H-S-H angle. The H_2_S molecule acquires a positive charge of 0.226 e, meaning that a charge transfer has occurred from the H_2_S molecule to the Be_12_O_12_ nano-cage. Figure 5b illustrates the H_2_S/Mg_12_O_12_ complex, while the H_2_S molecule dissociates into two fragments HS and H. The HS fragment is bonded via its S atom to a Mg site at a distance (d_1–25_) of 2.40 Å while the H fragment is attached to an O site at a distance (d_4–26_) of 0.98 Å. Due to that, an obvious deformation has occurred in Mg_12_O_12_ where the Mg-O bond length (d_1–4_) is elongated to 2.99 Å. The net acquired charge by the H_2_S molecule is −0.146 e, which means the Mg_12_O_12_ donates a charge to the H_2_S. Figure 5c demonstrates the SO_2_/Be_12_O_12_ complex, while the S atom of the SO_2_ molecule is bonded to an O site at a distance (d_2–25_) of 1.83 Å, and an O atom of the SO_2_ molecule is attached to a Be site at a distance (d_1–27_) of 1.57 Å. While for the SO_2_ molecule, the bond length (d_25–27_) is stretched to 1.56 Å and the O-S-O angle is slightly widened to 111.35°. In addition, the Be-O bond length (d_1–2_) is elongated to 2.62 Å. The SO_2_ molecule gains a negative charge of 0.127 e, i.e., a charge transfer has occurred from the Be_12_O_12_ nano-cage to the SO_2_ molecule. Furthermore, Figure 5d represents the SO_2_/Mg_12_O_12_ complex; it seems that three bonds are formed between the SO_2_ molecule and the Mg_12_O_12_ nano-cage. The two O atoms of the SO_2_ molecule are bonded to two Mg sites at distances of (d_3–27_) 1.98 Å and (d_7–26_) 2.06Å, while the S atom is attached to an O site at a distance (d_2–25_) of 1.64 Å. In addition, the two S-O bonds of the SO_2_ molecule are dilated to 1.54 Å while the O-S-O angle is diminished to 109.6° and the Mg-O bond length (d_2–3_) is elongated to 2.93 Å. Moreover, the SO_2_ molecule accepts a negative charge of −0.339 e; therefore, a charge transfer has occurred from the Mg_12_O_12_ nano-cage to the SO_2_ molecule. Additionally, the adsorption of H_2_S leads to a decrease in the HOMO–LUMO gap (E_g_) values of Be_12_O_12_ and Mg_12_O_12_ by 3.84% and 17.54%, respectively, whereas the adsorption of SO_2_ leads to a decrease in E_g_ values by 15.97% and 2.60%, respectively. Therefore, one can say that the E_g_ of the Be_12_O_12_ nano-cage is more sensitive to the SO_2_ than H_2_S adsorption while the E_g_ of the Mg_12_O_12_ nano-cage reveals higher sensitivity to H_2_S than SO_2_ adsorption.

The electrical conductivity (σ) and recovery time (τ) are important aspects of sensing applications. σ depends on E_g_ according to the following equation [72,73,74,75,76,77]:

(10) $$\Delta G=G_{\mathrm{gas}/\mathrm{nano}-\mathrm{cage}}-\left(G_{\mathrm{gas}}+G_{\mathrm{nano}-\mathrm{cage}}\right)$$

where A is a constant, k is Boltzmann’s constant, and T is the temperature. Therefore, the increase of σ value of the Be_12_O_12_ nano-cage in the presence of SO_2_ gas is higher than in the presence of H_2_S gas, while the increase of σ value of the Mg_12_O_12_ nano-cage in the presence of H_2_S gas is higher than in the presence of SO_2_ gas. The τ is related to the E_ads_ as in Equation (11) [78,79]:

(11) $$\tau =v_{o}^{-1}\exp\left(\frac{-E_{\mathrm{ads}}}{\mathrm{KT}}\right)$$

where $\nu_{o}$ is the attempt frequency. In other words, as E_ads_ increases (more negative) the longer  τ becomes. Therefore, in the obtained E_ads_ values, τ trends as follows: SO_2_/Mg_12_O_12_ > H_2_S/Mg_12_O_12_ > SO_2_/Be_12_O_12_ > H_2_S/Be_12_O_12_. Thus, our results may be fruitful for sensing applications.

To illuminate the features of the interaction between the considered gases and the sorbent nano-cages, our results will be discussed related to the following: (i) NBO atomic charges as well charge density difference analysis (Δρ), (ii) bond analysis, and (iii) PDOS analysis.

#### 3.2.1. NBO and Charge Density Difference Analysis

To shed light on the mechanism of H_2_S and SO_2_ interaction with the considered nano-cages, NBO analysis, as well as charge density difference (Δρ) analysis, has been performed. Table 3 lists the atomic NBO charges, as well the electronic configuration of the atoms, for the free H_2_S, Be_12_O_12_, Mg_12_O_12_, H_2_S/Be_12_O_12_, and H_2_S/Mg_12_O_12_. The numbering of atoms as shown in Figure 5 is used.

For the H_2_S/Be_12_O_12_ structure, it is clear that due to the interaction, the 1s orbital of the H26 and H27 atoms loses charges of 0.03 and 0.02 e while the 3s and 3p orbitals of the S atom lose charges of 0.03 and 0.14 e, respectively. On the other hand, the 2s and 2p orbitals of the Be1 atom gain charges of 0.14 and 0.04 e while for the O2, O4, and O6 the 2s orbital loses a charge of 0.01e whereas the 2p orbital gains a charge of 0.01 e. This explains why a total charge of 0.226 e, as shown in Table 2, has been transferred from H_2_S to the Be_12_O_12_ nano-cage and the major of the charge transfer has occurred from the S atom of H_2_S to the Be1 atom of the Be_12_O_12_ nano-cage. 

**Figure 6:**
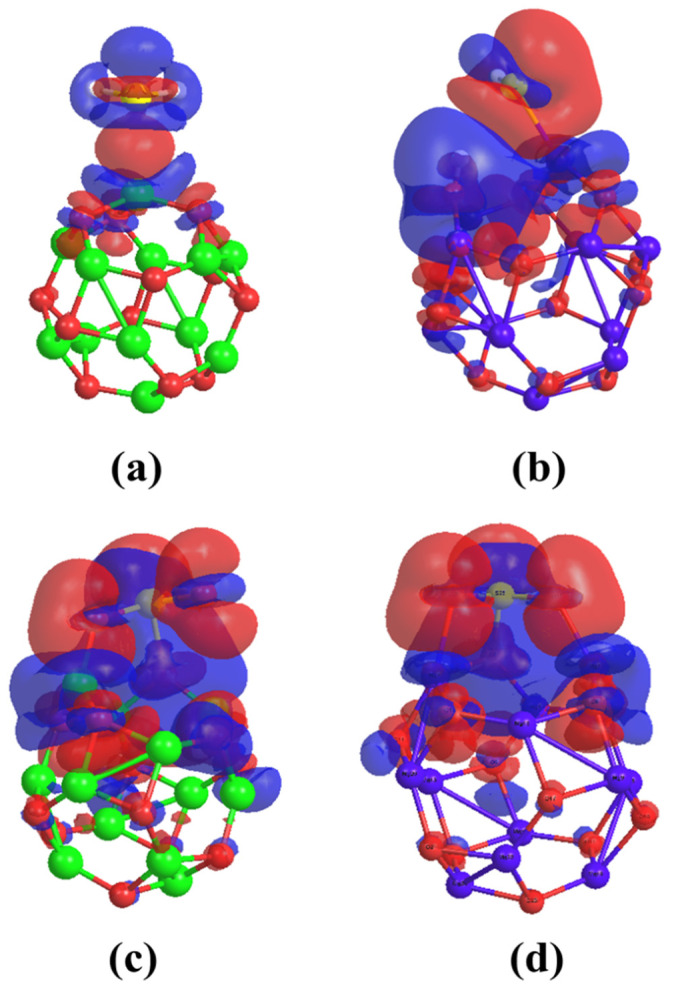
Charge density difference (Δρ) for (**a**) H_2_S/Be_12_O_12_, (**b**) H_2_S/Mg_12_O_12_, (**c**) SO_2_/Be_12_O_12_, and (**d**) SO_2_/Mg_12_O_12_ complexes. Red color for negative Δρ and blue color for positive Δρ.

This means the major mechanism of the interaction is the charge transfer mechanism. In addition, a slight loss and gain of charges are observed simultaneously for the O2, O4, and O6; therefore, one can suggest another minor mechanism which is the donation–back donation mechanism. Figure 6a demonstrates Δρ for H_2_S/Be_12_O_12_ complex.

The H and S atoms of the H_2_S molecule are surrounded by positive Δρ values (blue color), which confirms the charge transfer from the H_2_S molecule to the nano-cage. In addition, the positive (blue color) and negative (red color) Δρ values around each of the O2, O4, O6, and S atoms confirm the donation–back donation mechanism. For H_2_S/Mg_12_O_12_ structure, the interaction between H_2_S and Mg_12_O_12_ leads to the following: the 1s orbital of the H26 loses a charge of 0.37 e whereas the 2s and 2p of the O4 atom lose a charge of 0.07 and 0.13 e, respectively. Furthermore, the H27 atom has no change, the 3s and 3p orbitals of the S atom gain charges of 0.05 and 0.49 e, and the 3s and 3p of the Mg gain charges of 0.06 and 0.06 e, respectively. This confirms the dissociation of the H_2_S molecule into H^+^ and SH^−^. Then, the H^+^ is attached to the O4 atom, while the SH^−^ is attached to the Mg1 atom. Figure 6b shows Δρ for the H_2_S/Mg_12_O_12_ complex. The H26 is surrounded by positive Δρ values (blue color) while the S atom is surrounded by negative (red color) Δρ values which agree with the above discussion. Table 4 illustrates the atomic NBO charges, as well the electronic configuration of the atoms, for the free SO_2_, Be_12_O_12_, Mg_12_O_12_, SO_2_/Be_12_O_12_, and SO_2_/Mg_12_O_12_.

For SO_2_/Be_12_O_12_ structure, one can observe, that the 2s of the O27 and the 3s and 3p of the S atom lose charges of 0.08, 0.07, and 0.10 e, respectively, while the 2p of the O26 and O27 atoms gain charges of 0.11 and 0.32 e, respectively. Therefore, the positive charge of the S atom and the negative charge of the O27 atom increase; consequently, they are attached to the O2 negative and Mg1 positive sites of the nano-cage, respectively. Moreover, for the Be1 atom, the 2s gains a charge of 0.01 e while the 2p loses a charge of 0.04 e, whereas for the O2 atom, the 2s gains a charge of 0.06 e while the 2p orbital loses a charge of 0.13 e. Therefore, one can say that there is a charge transfer from the Be_12_O_12_ nano-cage to the SO_2_ molecule greater than the charge transferred from the SO_2_ molecule to the Be_12_O_12_ nano-cage. This explains why SO_2_ has a total charge of −0.127 e, as shown in Table 2. Δρ for the SO_2_/Be_12_O_12_ complex is demonstrated in Figure 6c. It is clear that both the adsorbed SO_2_ molecule and the sorbent Be_12_O_12_ nano-cage are surrounded by positive and negative Δρ values (blue and red colors) which confirms the donation–back donation mechanism for the interaction. For the SO_2_/Mg_12_O_12_ structure, one can notice that the 2s of the O26 and O27 loses charges of 0.02 and 0.03 e while the 2p gains charges of 0.28 and 0.34 e, respectively. Moreover, the 3s and 3p of the S atom lose charges of 0.11 and 0.06 e, respectively. Therefore, the net atomic charges of the O atoms of the SO_2_ become more negative while the S atom becomes more positive, consequently, the O26 and O27 are attracted to the Mg7 and Mg3 positive sites while the S atom is attracted to the O2 negative site of the Mg_12_O_12_ nano-cage, whereas for the Mg_12_O_12_, the 2s and 2p of the O2 atom lose charges of 0.02 and 0.28 e while the rest atoms of the nano-cage have little gains and loss of charges. Δρ for the SO_2_/Mg_12_O_12_ complex is illustrated in Figure 6d. It is clear that the adsorbed SO_2_ molecule is surrounded by negative Δρ values greater than the positive Δρ values, which confirm the donation–back donation mechanism for the interaction.

#### 3.2.2. Bond Analysis

Bond order and overlap population are estimated for the free adsorbed gasses as well gas/nano-cage complexes. As the overlap value decreases, the interaction between the two atoms decreases and vice versa whereas the values close to zero mean no interaction while overlapping positive and negative values indicate the bonding and anti-bonding states, respectively [71,75,80]. Table 5 concerns the free H_2_S molecule, H_2_S/Be_12_O_12_, and H_2_S/Mg_12_O_12_ complexes.

For H_2_S/Be_12_O_12,_ the overlap population and bond order values of the S-H26 and S-H27 bonds are slightly changed with respect to the free H_2_S molecule, while overlapping population and bond order values of 0.074 and 0.279, respectively, are observed for the S-Be1 bond. This indicates the formation of a weak bond between the H_2_S molecule and the B_12_O_12_ nano-cage. On the other hand, for H_2_S/Mg_12_O_12_, the low overlap population and bond order values for the S-H26 bond indicate the dissociation of the H_2_S molecule. In addition, high overlapping population and bond order values for S-Mg1 and O4-H26 indicate bond formation between the S atom and the Mg1 atom and between the H26 atom and the O4 atom. Furthermore, the overlapping population and the bond order values for the Mg1-O4 are decreased to 0.035 and 0.106 rather than 0.183 and 0.520 for the bare nano-cage, respectively, indicating a bond weakness has occurred. This reveals the strong interaction between the H_2_S molecule and the Mg_12_O_12_ nano-cage. Table 6 is interested in the free SO_2_ molecule, SO_2_/Be_12_O_12_, and SO_2_/Mg_12_O_12_ complexes.

For SO_2_/Be_12_O_12_, the S-O27 and Be1-O2 bonds are weakened as indicated by the low values of the overlap population and bond order, while the high overlap population and bond order values for the Be1-O27 bond indicate bond formation. On the other hand, for SO_2_/Mg_12_O_12_, the decrease in the S-O26, S-O27, and Mg3-O2 overlapping population and bond order values indicates the weakness of these bonds while the bond order of 0.892, 0.464, and 0.495 for the S-O2, Mg-O27, and Mg-O26, respectively, confirms the formation of these bonds. In other words, one bond is formed between the SO_2_ and the nano-cage for the SO_2_/Be_12_O_12_ complex, whereas three bonds are formed for the SO_2_/Mg_12_O_12_ complex. This explains the higher adsorption energy for SO_2_/Mg_12_O_12_ than SO_2_/Be_12_O_12_.

#### 3.2.3. PDOS Analysis

**Figure 7:**
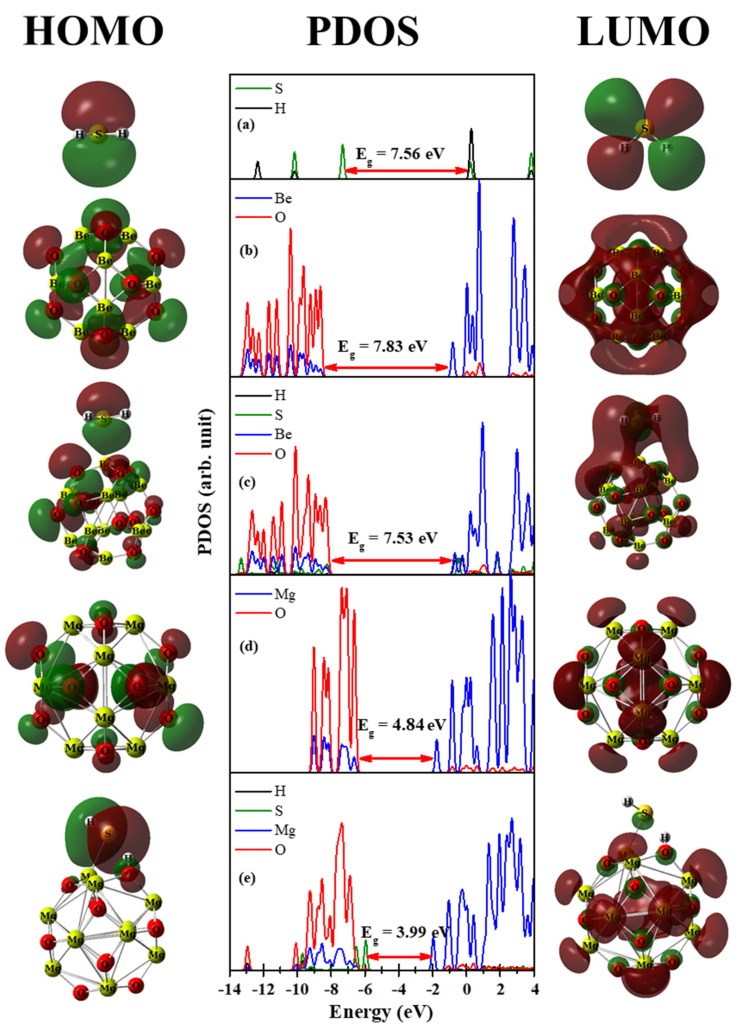
HOMO, PDOS, LUMO for (**a**) H_2_S, (**b**) Be_12_O_12_, (**c**) H_2_S/Be_12_O_12_, (**d**) Mg_12_O_12_, and (**e**) H_2_S/Mg_12_O_12_ complexes.

Figure 7 illustrates the surfaces of the HOMO and LUMO as well as the PDOS for the free H_2_S molecule, the bare Be_12_O_12_, and Mg_12_O_12_ nano-cages, as well the H_2_S/Be_12_O_12_ and H_2_S/Mg_12_O_12_ complexes.

**Figure 8:**
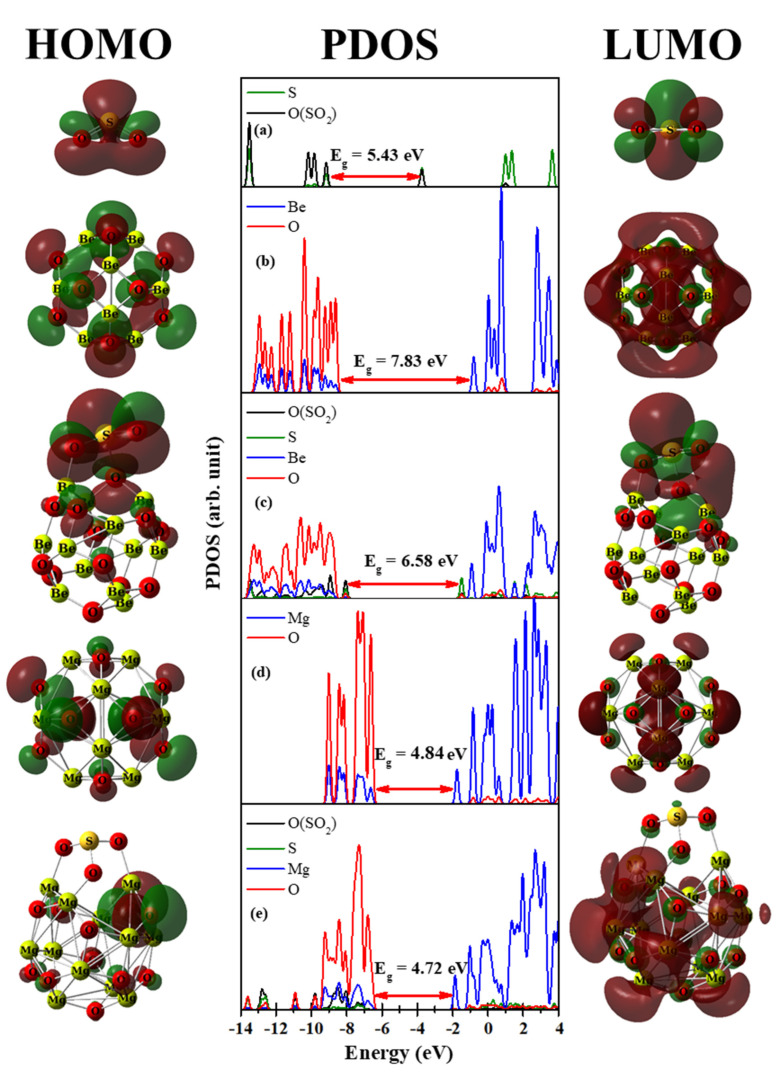
HOMO, PDOS, LUMO for (**a**) SO_2_, (**b**) Be_12_O_12_, (**c**) SO_2_/Be_12_O_12_, (**d**) Mg_12_O_12_, and (**e**) SO_2_/Mg_12_O_12_ complexes.

Figure 7a shows three occupied states for the H_2_S molecule at −12.36, −10.12, and −7.30 eV; the H atom states located at −12.36 and −10.12 eV; and the S atom states located at −12.36, −10.12, and −7.35 eV. The S atom states for free H_2_S molecule are disappeared in the H_2_S/Be_12_O_12_ complex, as shown in Figure 7c. Furthermore, new states for the S atom are observed in the H_2_S/Be_12_O_12_ complex; these states overlap with the Be and O states, which emphasizes the interaction between the H_2_S molecule with the nano-cage. For instance, the states of the S, Be, and O atoms of the complex are overlapped at −8.20 eV, which is the HOMO of the complex. The HOMO surface of the complex displays the contribution of the S, Be, and O atoms. Comparing Figure 7b,c, one can observe the adsorption of the H_2_S molecule rises the HOMO and LUMO of Be_12_O_12_ by 0.43 and 0.13 eV, respectively; therefore, a decrease of 0.30 eV in the HOMO–LUMO gap has been recorded. For H_2_S/Mg_12_O_12_ complex, Figure 7e, it is clear that there is an overlap between the H and O states at −12.92 eV, which is due to the interaction between H26 and O4 in the complex. Moreover, the occupied states of the S atom are located at −9.70, −6.51, and −5.93 eV, i.e., they are shifted up with respect to the free H_2_S, which confirms the strong interaction between the H_2_S and Mg_12_O_12_ nano-cage. In addition, comparing Figure 7d,e, one can see that HOMO rises by 1.02 eV while LUMO lowers by 0.19 eV. Thus, H_2_S adsorption narrows the HOMO–LUMO gap by 0.85 eV. It is worth noticing that only the states of S and O atoms appear in the HOMO states, and this agrees with the obtained HOMO surface for the H_2_S/Mg_12_O_12_ complex. Figure 8 demonstrates the surfaces of HOMO and LUMO, as well PDOS, for the free SO_2_ molecule, bare Be_12_O_12_, and Mg_12_O_12_ nano-cages, as well SO_2_/Be_12_O_12_ and SO_2_/Mg_12_O_12_ complexes.

Comparing Figure 8a–c, it is clear that there are dramatic changes in the states due to the adsorption of the SO_2_ molecule on the Be_12_O_12_ nano-cage, where the states of SO_2_ overlap with the states of Be_12_O_12_. For example, one can observe the appearance of the S atom and the O atoms of the SO_2_ and Be_12_O_12_ in the HOMO states of the SO_2_/Be_12_O_12_ at −8.05 eV. This is confirmed by the HOMO surface for the SO_2_/Be_12_O_12_ complex. Based on the interaction, the HOMO of the nano-cage rises by 0.57 eV and the LUMO lowers by 0.68 eV, in turn, narrowing the HOMO–LUMO gap by 1.25 eV. Comparing Figure 8a,d,e, one can observe that adsorption of the SO_2_ molecule on the Mg_12_O_12_ nano-cage leads to intense changes in the states of the SO_2_ as well as the states of the Mg_12_O_12,_ which confirms the occurrence of a strong interaction. Moreover, the HOMO of the nano-cage increases by 0.04 while the LUMO decreases by 0.08 eV; consequently, the HOMO–LUMO gap is slightly decreased by 0.12 eV.

### 3.3. Molecular Dynamic Simulations

**Figure 9:**
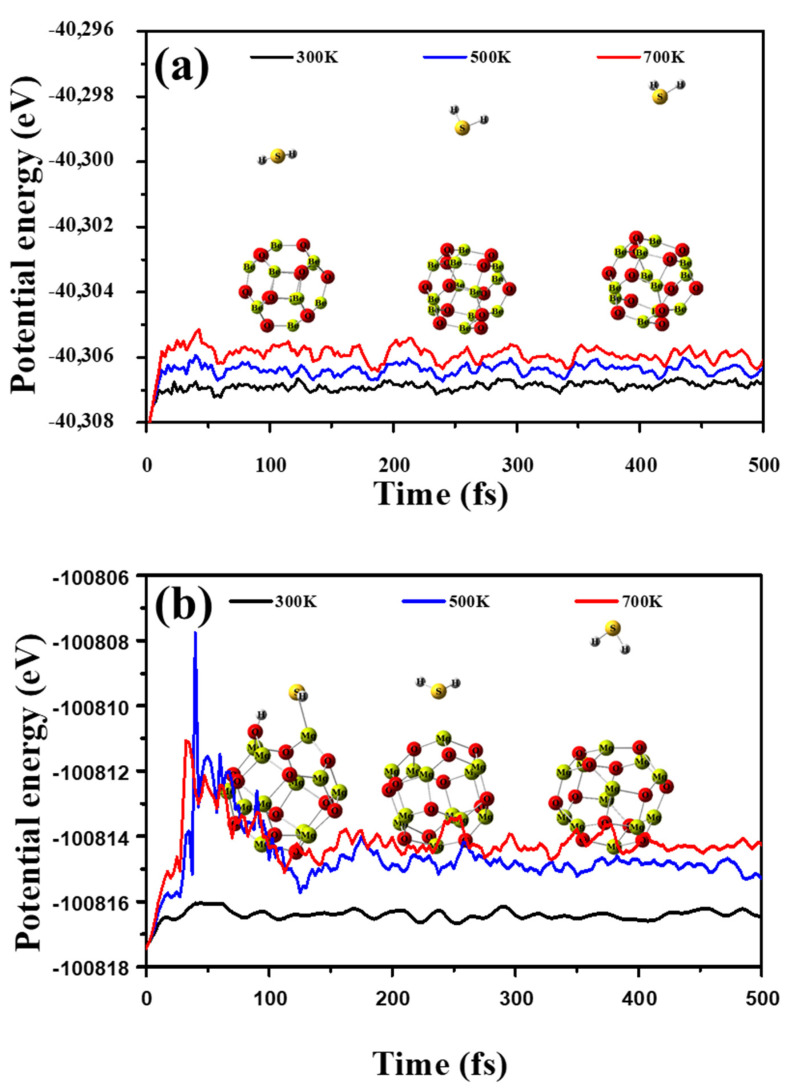
Potential energy fluctuations during MD simulation for (**a**) H_2_S/Be_12_O_12_, and (**b**) H_2_S/Mg_12_O_12_ and the atomic configuration after 500 fs at 300, 500, and 700 K.

To examine the impact of the temperature on the adsorption process of the investigated gases, molecular dynamic (MD) simulations at 300, 500, and 700 K for a total time of 500 fs are performed for H_2_S/Be_12_O_12_, H_2_S/Mg_12_O_12_, SO_2_/Be_12_O_12_, and SO_2_/Mg_12_O_12_ complexes. MD simulations are carried out via the ADMP model. Figure 9a,b illustrates the potential energy fluctuations for H_2_S/Be_12_O_12_ and H_2_S/Mg_12_O_12_, respectively, as well as the atomic configuration after 500 fs at the inspected temperatures.

**Figure 10:**
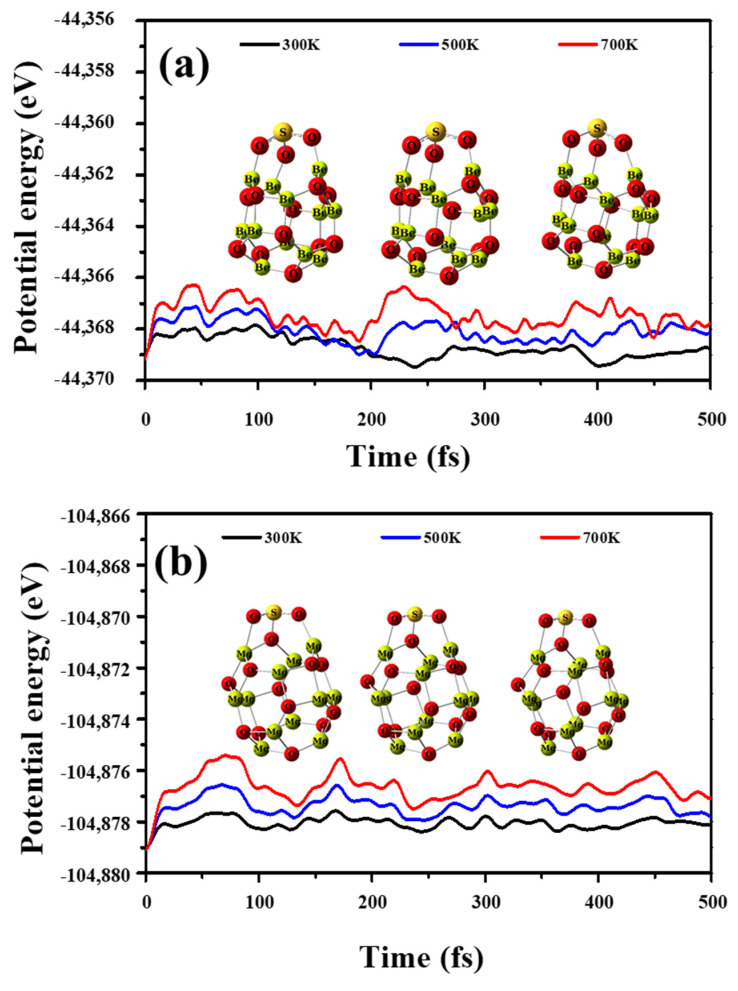
Potential energy fluctuations during MD simulation for (**a**) SO_2_/Be_12_O_12_, and (**b**) SO_2_/Mg_12_O_12_ and the atomic configuration after 500 fs at 300, 500, and 700 K.

For H_2_S/Be_12_O_12,_ Figure 9a, although the fluctuation of the potential energy is small, the distance (d_1–25_) between H_2_S and Be_12_O_12_ nano-cage increased with time. As well as the temperature increases, the increment in the distance increases, where the d_1–25_ values at the end of the time increase to 3.32, 7.38, and 9.55 Å for temperatures 300, 500, and 700 K, respectively. Therefore, one suggests that the Be_12_O_12_ nano-cage cannot retain H_2_S on its surface, especially at high temperatures. For H_2_S/Mg_12_O_12_, Figure 9b, at the temperature of 300 K, the fluctuation of the potential energy is small, and the Mg_12_O_12_ nano-cage preserves the dissociated H_2_S molecule on its surface with no significant changes in the geometrical structure of the complex, while at the temperatures of 500 and 700 K, a high fluctuation of the potential energy is observed until 120–130 fs, when the fluctuation decreases. In addition, the dissociation of the H_2_S molecule is diminished. At the end of the time, at 500 K, the H_2_S is retained on the Mg_12_O_12_ nano-cage at a distance of 2.65 Å while at 700 K the d_1–25_ increases to 6.79 Å. Furthermore, Figure 10a,b demonstrates the potential energy fluctuations for SO_2_/Be_12_O_12_ and SO_2_/Mg_12_O_12_, respectively, as well as the atomic configuration after 500 fs at the inspected temperatures.

It is clear that no significant fluctuation of the potential energy is observed. Moreover, at the end of the time, there is a trivial deformation in the geometrical structure of the SO_2_/Be_12_O_12_ and SO_2_/Mg_12_O_12_ complexes. Therefore, one proposes that Be_12_O_12_ and Mg_12_O_12_ nano-cages can retain the SO_2_ molecule on their surface at temperatures up to 700 K.

### 3.4. Thermodynamic Properties

**Figure 11:**
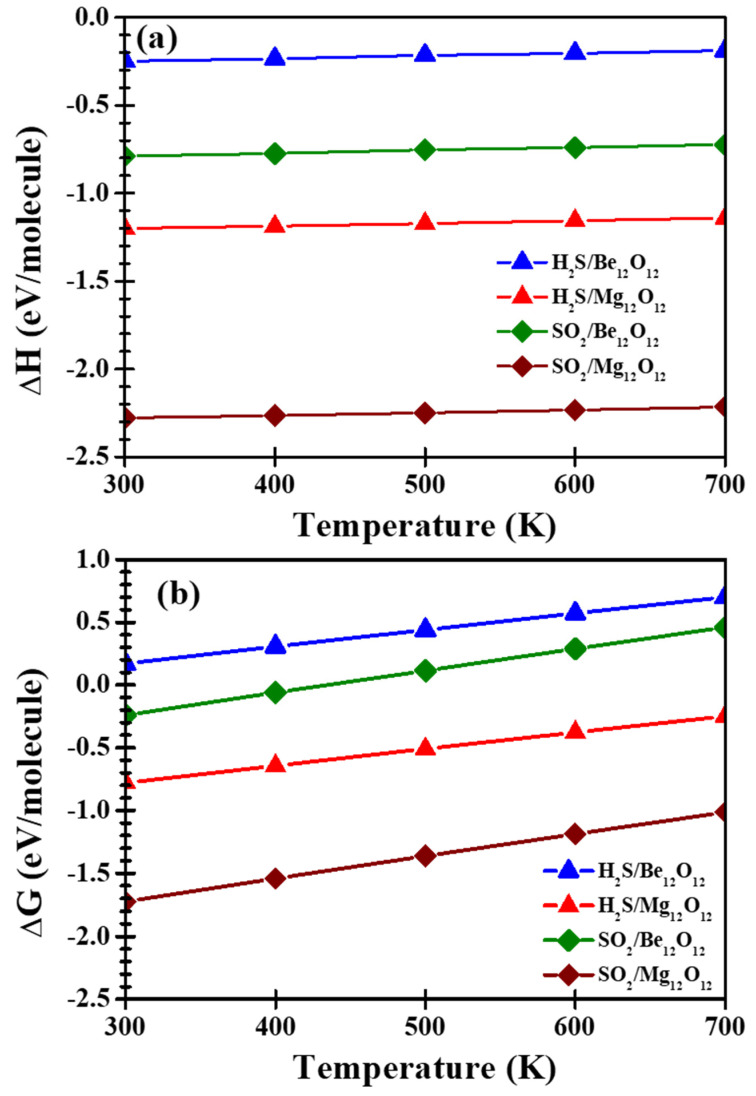
(**a**) ΔH and (**b**) ΔG for H_2_S/Be_12_O_12_, H_2_S/Mg_12_O_12_, SO_2_/Be_12_O_12_, and SO_2_/Mg_12_O_12_ complexes.

For gas adsorption, enthalpy difference (ΔH) and free energy difference (ΔG) are imperative thermodynamic parameters for determining the strength and the spontaneity of the reaction. Therefore, thermodynamic calculations for H_2_S/Be_12_O_12_, H_2_S/Mg_12_O_12_, SO_2_/Be_12_O_12_, and SO_2_/Mg_12_O_12_ complexes have been performed in the temperature range 300–700 K. Figure 11a signifies ΔH for the investigated complexes.

ΔH values for all considered complexes are negative, which specifies the reactions between H_2_S as well SO_2_ with Be_12_O_12_ and Mg_12_O_12_ nano-cages are exothermic. Furthermore, as the temperature increases, the negative ΔH values decrease, which indicates the reactions are stronger at lower temperatures. In addition, for the same gas, ΔH values are more negative for the Mg_12_O_12_ nano-cage than the Be_12_O_12_ nano-cage, while for the same nano-cage, ΔH values are more negative for SO_2_ gas than H_2_S gas. This confirms the above discussion of the high ability of the Mg_12_O_12_ nano-cage to absorb the investigated gases and the high ability of the SO_2_ gas to attach to the considered nano-cages. Figure 11b shows ΔG for the examined complexes. Spontaneous and non-spontaneous reactions are characterized by negative and positive ΔG values, respectively, while low negative ΔG values indicate the capability to reverse the reaction [46,66,67,68]. For the H_2_S/Be_12_O_12_ complex in the temperature range, ΔG values are positive, which indicates a non-spontaneous reaction, while for the SO_2_/Be_12_O_12_ complex, the reaction is spontaneous at low temperatures, and beyond T = 400 K, the reaction turns into a non-spontaneous reaction. Furthermore, for the H_2_S/Mg_12_O_12_ and SO_2_/Mg_12_O_12_ complexes in the temperature range, ΔG values are negative, which indicates a spontaneous reaction. In addition, the reaction is capable of being reversed in the H_2_S/Mg_12_O_12_ complex easier than in the SO_2_/Mg_12_O_12_ complex.

## 4. Conclusions

Structural and electronic properties of the considered Be_12_O_12_ and Mg_12_O_12_ nano-cages as well their stability are scrutinized. Mg_12_O_12_ exhibits lower E_g_, IP, η, and higher ω values than those for Be_12_O_12;_ therefore, Mg_12_O_12_ is more reactive than the Be_12_O_12_ nano-cage. Molecular dynamics calculations emphasize the stability of the investigated nano-cages. In addition, the interaction of H_2_S and SO_2_ gases at the surfaces of the inspected nano-cages have been studied, and the features of the interaction are examined in the point of the NBO atomic charges, charge density difference analysis (Δρ), bond analysis, and PDOS. $E_{\mathrm{ads}}^{\mathrm{corr}}$ values show that the ability of the Mg_12_O_12_ nano-cage to adsorb the considered gases is higher than that of the Be_12_O_12_ nano-cage. Furthermore, the ability of SO_2_ gas to be adsorbed is higher than that of H_2_S gas. Furthermore, H_2_S gas dissociates at the Mg_12_O_12_ surface. In addition, adsorption of H_2_S leads to a decrease in the HOMO–LUMO gap (E_g_) values of Be_12_O_12_ and Mg_12_O_12_ by 3.84% and 17.54%, respectively, whereas the adsorption of SO_2_ leads to a decrease in E_g_ values by 15.97% and 2.60%, respectively. At high temperatures, MD calculations declare that the Be_12_O_12_ and Mg_12_O_12_ nano-cages do not retain the H_2_S on their surfaces, while SO_2_ is retained at low and high temperatures. Moreover, the thermodynamic calculations show that the reactions between H_2_S and SO_2_ with Be_12_O_12_ and Mg_12_O_12_ nano-cages are exothermic. Furthermore, at the temperature range of 300–700 K, the H_2_S reaction with Mg_12_O_12_ and Be_12_O_12_ is spontaneous and non-spontaneous, respectively, while the SO_2_ reaction with Mg_12_O_12_ is spontaneous, whereas the SO_2_ reaction with Be_12_O_12_ is spontaneous at temperatures up to 400 K. In addition, the reaction is capable to be reversed in the H_2_S/Mg_12_O_12_ complex easier than in the SO_2_/Mg_12_O_12_ complex.

## Figures and Tables

**Table 1:** Electronic properties of Be_12_O_12_ and Mg_12_O_12_ nano-cages. HOMO and LUMO energy levels (eV), HOMO- LUMO gap (E_g_, eV), ionization potential (IP, eV), NBO charges (Q, e), chemical potential (**µ**, eV), hardness (**η**, eV), electrophilicity (**ω**, eV), and dipole moment (D, Debye).

	Be_12_O_12_		Mg_12_O_12_	
	Present Study	Previous Studies	Present Study	Previous Studies
HOMO	−8.62	−8.62 [57]	−6.59	−6.58 [57], −6.57 [58], −6.60 [38], −6.58 [59],
				−6.74 [30,31], −6.53 [10]
LUMO	−0.79	−1.07 [57]	−1.75	−1.78 [57], −1.71 [58], −1.80 [38], −1.72 [59]
E_g_	7.83	8.29 [43], 7.41 [45], 7.55 [57]	4.84	4.87 [43], 4.83 [45], 4.79 [57], 4.86 [58,59], 4.78 [30,31]
IP	10.27		7.98	
Q_M_	1.19		1.44	
Q_O_	−1.19		−1.44	
µ	−4.71		−4.17	−4.14 [58]
η	3.92		2.42	
ω	2.83		3.60	
D	0.00		0.01	0.01 [58], 0.00 [38], 0.07 [59]

M = Be and Mg.

**Table 2:** Adsorption properties of H_2_S as well SO_2_ on Be_12_O_12_ and Mg_12_O_12_ nano-cages. Adsorption energies (E_ads_, eV), Basis set superposition error (BSSE, eV), corrected adsorption energy ($E_{\mathrm{ads}}^{\mathrm{corr}}$, eV), HOMO and LUMO energy levels (eV), HOMO- LUMO gap (E_g_, eV), NBO charges (Q, e), and dipole moment (D, Debye).

	**H_2_S**	**H_2_S/Be_12_O_12_**	**H_2_S/Mg_12_O_12_**
E_ads_	-	−0.31 (−0.50)	−1.32 (−1.57)
BSSE	-	0.05 (0.05)	0.09 (0.10)
$E_{\mathrm{ads}}^{\mathrm{corr}}$	-	−0.26 (−0.45)	−1.23 (−1.47)
HOMO	−7.31 (−7.31)	−8.20 (−8.22)	−5.93 (−5.95)
LUMO	0.26 (0.26)	−0.67 (−0.68)	−1.94 (−1.93)
E_g_	7.56 (7.56)	7.53 (7.54)	3.99 (4.02)
$Q_{H_{2}S}$	0.00 (0.00)	0.23 (0.24)	−0.15 (−0.16)
D	1.33 (1.33)	3.27 (3.22)	5.50 (5.48)
	**SO_2_**	**SO_2_/Be_12_O_12_**	**SO_2_/Mg_12_O_12_**
E_ads_	-	−0.83 (−1.07)	−2.33 (−2.52)
BSSE	-	0.38 (0.39)	0.41 (0.41)
$E_{\mathrm{ads}}^{\mathrm{corr}}$	-	−0.45 (−0.68)	−1.93 (−2.11)
HOMO	−9.16 (−9.16)	−8.05 (−8.05)	−6.55 (−6.55)
LUMO	−3.73 (−3.73)	−1.47 (−1.49)	−1.84 (−1.83)
E_g_	5.43 (5.43)	6.58 (6.57)	4.71 (4.72)
$Q_{SO_{2}}$	0.00 (0.00)	−0.13 (−0.13)	−0.34 (−0.34)
D	1.94 (1.94)	2.95 (2.83)	4.59 (4.58)

Values between brackets are calculated at B3LYP/6–311g(d,p) with dispersion correction (DFT-D3).

**Table 3:** NBO charges (Q, e) and electronic configuration for free H_2_S, Be_12_O_12_, Mg_12_O_12_, H_2_S/Be_12_O_12_, and H_2_S/Mg_12_O_12_.

Structure	Atom	Atomic Charge	Electronic Configuration				
			1s	2s	2p	3s	3p
H_2_S	H	0.134	0.86	-	-	-	-
	S	−0.268	-	-	-	1.76	4.48
Be_12_O_12_	Be	1.186	-	0.21	0.59	-	-
	O	−1.186	-	1.69	5.49	-	-
Mg_12_O_12_	Mg	1.443	-	-	-	0.21	0.35
	O	−1.443	-	1.84	5.60	-	-
H_2_S/Be_12_O_12_	H26	0.160	0.83	-	-	-	-
	H27	0.159	0.84	-	-	-	-
	S	−0.093	-	-	-	1.73	4.34
	Be1	1.007	-	0.25	0.73	-	-
	O2	−1.191	-	1.68	5.50	-	-
	O4	−1.189	-	1.68	5.50	-	-
	O6	−1.190	-	1.68	5.50	-	-
H_2_S/Mg_12_O_12_	H26	0.509	0.49	-	-	-	-
	H27	0.130	0.86	-	-	-	-
	S	−0.785	-	-	-	1.81	4.97
	Mg1	1.305	-	-	-	0.27	0.41
	O2	−1.452	-	1.83	5.62	-	-
	O4	−1.251	-	1.77	5.47	-	-
	O6	−1.442	-	1.83	5.61	-	-

**Table 4:** NBO charges (Q, e) and electronic configuration for free SO_2_, Be_12_O_12_, Mg_12_O_12_, SO_2_/Be_12_O_12_, and SO_2_/Mg_12_O_12_.

Structure	Atom	Atomic Charge	Electronic Configuration			
			2s	2p	3s	3p
SO_2_	O	−0.747	1.84	4.89	-	-
	S	1.495	-	-	1.62	2.68
Be_12_O_12_	Be	1.186	0.21	0.59	-	-
	O	−1.186	1.69	5.49	-	-
Mg_12_O_12_	Mg	1.443	-	-	0.21	0.35
	O	−1.443	1.84	5.60	-	-
SO_2_/Be_12_O_12_	O26	−0.852	1.84	5.00	-	-
	O27	−0.980	1.76	5.21	-	-
	S	1.705	-	-	1.55	2.58
	Be1	1.210	0.22	0.55	-	-
	O2	−1.109	1.75	5.36	-	-
	O4	−1.214	1.69	5.52	-	-
	O6	−1.197	1.69	5.50	-	-
SO_2_/Mg_12_O_12_	O26	−1.003	1.82	5.17	-	-
	O27	−1.049	1.81	5.23	-	-
	S	1.713	-	-	1.51	2.62
	Mg1	1.477	-	-	0.20	0.32
	Mg3	1.468	-	-	0.20	0.32
	Mg7	1.417	-	-	0.21	0.36
	O2	−1.148	1.82	5.32	-	-
	O4	−1.463	1.84	5.62	-	-
	O8	−1.454	1.84	5.61	-	-

**Table 5:** Overlap population and bond order of H_2_S, H_2_S/Be_12_O_12_, and H_2_S/Mg_12_O_12_.

Structure	H_2_S		H_2_S/Be_12_O_12_		H_2_S/Mg_12_O_12_	
	Overlap Pop.	Bond Order	Overlap Pop.	Bond Order	Overlap Pop.	Bond Order
S–H26	0.304	0.979	0.316	0.974	0.042	0.097
S–H27	0.304	0.979	0.313	0.972	0.266	0.956
S–M1	-	-	0.074	0.279	0.273	0.717
O4–H26	-	-	0.000	0.002	0.289	0.835
M1–O4	-	-	0.206 (0.220) *	0.538 (0.602) *	0.035 (0.183) *	0.106 (0.520) *

* Values between brackets are for the bare nano-cage. M = Be and Mg.

**Table 6:** Overlap population and bond order of SO_2_, SO_2_/Be_12_O_12_, and SO_2_/Mg_12_O_12_.

Structure	SO_2_		SO_2_/Be_12_O_12_		SO_2_/Mg_12_O_12_	
	Overlap Pop.	Bond Order	Overlap Pop.	Bond Order	Overlap Pop.	Bond Order
S-O26	0.242	1.773	0.302	1.706	0.203	1.290
S-O27	0.242	1.773	0.116	1.138	0.156	1.219
S-O2	-	-	−0.015	0.564	0.079	0.892
M1-O27	-	-	0.262	0.614	0.000	−0.010
M3-O27	-	-	0.022	0.022	0.192	0.464
M7-O26	-	-	0.057	0.118	0.207	0.495
M1-O2	-	-	−0.006 (0.220) *	0.028 (0.602) *	0.113 (0.183) *	0.267 (0.520) *
M3-O2	-	-	0.215 (0.220) *	0.452 (0.602) *	0.033 (0.183) *	0.103 (0.520) *

* Values between brackets are for the bare nano-cage. M = Be and Mg.

## Data Availability

Not applicable.

## Supplementary Materials

The following supporting information can be downloaded at: https://www.mdpi.com/article/10.3390/nano12101757/s1, Table S1. The examined orientations for H_2_S interaction with Be_12_O_12_, Table S2. The examined orientations for H_2_S interaction with Mg_12_O_12_, Table S3. The examined orientations for SO_2_ interaction with Be_12_O_12_, Table S4. The examined orientations for SO_2_ interaction with Mg_12_O_12_.
