# Supplementary material for: Mg12O12 and Be12O12 Nanocages as Sorbents and Sensors for H2S and SO2 Gases: A Theoretical Approach
Source: Nanomaterials (Basel). 2022 May 21;12(10):1757. doi: 10.3390/nano12101757 (PMC9143161; doi:10.3390/nano12101757)
Supplement: Supplementary file 1 [file nanomaterials-12-01757-s001.zip › nanomaterials-1716591-supplementary.pdf]

Supplementary Materials

$Mg_{12}O_{12}$ and $Be_{12}O_{12}$ Nanocages as Sorbents and Sensors for H_2S and SO_2 Gases: A Theoretical Approach

H. M. Badran [1], Kh. M. Eid [2,3], Sotirios Baskoutas [4] and H. Y. Ammar [1,*]

[1] Physics Department, College of Science and Arts, Najran University, Najran 11001, Saudi Arabia; hmbadran@nu.edu.sa
[2] Physics Department, Faculty of Education, Ain Shams University, Cairo 11566, Egypt; khmeid@yahoo.com
[3] Department of Physics, College of Science and Arts, Qassim University, Albukayriyah 52725, Saudi Arabia
[4] Department of Materials Science, University of Patras, Patras 26504, Greece; bask@upatras.gr
* Correspondence: hyammar@hotmail.com

Table S1. The examined orientations for H_2S interaction with $Be_{12}O_{12}$.

mode	Input structure	Optimized structure	mode	Input structure	Optimized structure
1	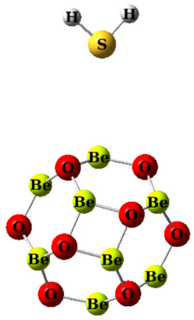	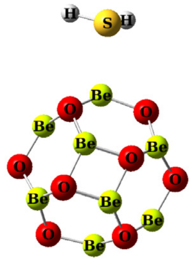	5	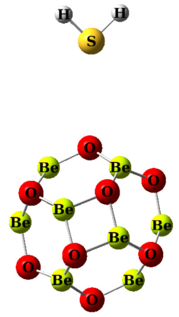	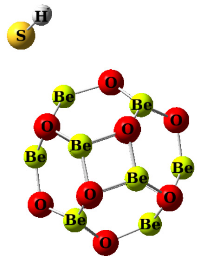
	E_{ads} = -0.31 eV			E_{ads} = -0.30 eV	
2	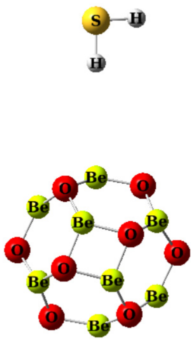	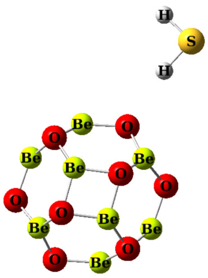	6	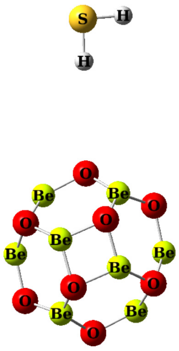	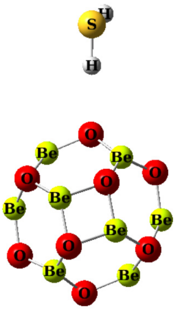
	E_{ads} = -0.08 eV			E_{ads} = -0.09 eV	

mode	Input structure	Optimized structure	mode	Input structure	Optimized structure
3	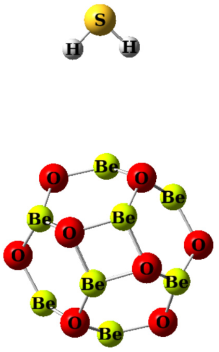	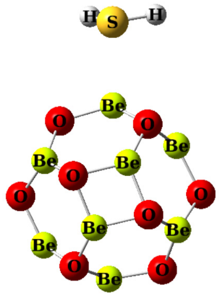 E_{ads} = -0.31 eV	7	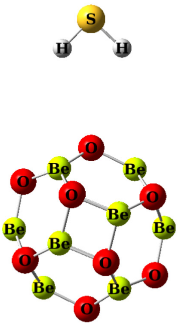	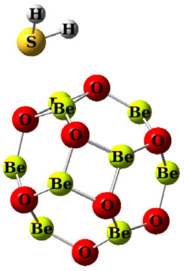 E_{ads} = -0.31 eV
4	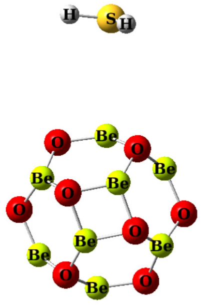	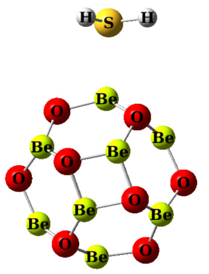 E_{ads} = -0.31 eV	8	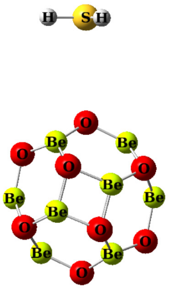	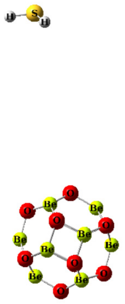 E_{ads} = -0.01 eV

Table S2. The examined orientations for H_2S interaction with $Mg_{12}O_{12}$.

mode	Input structure	Optimized structure	mode	Input structure	Optimized structure
1	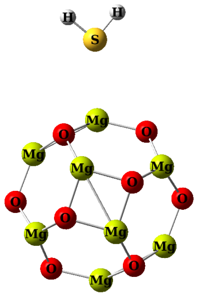	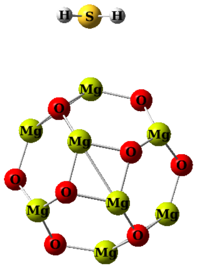 E_{ads} = -0.40 eV	5	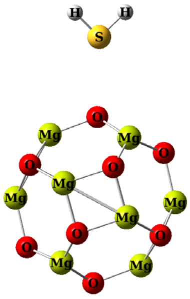	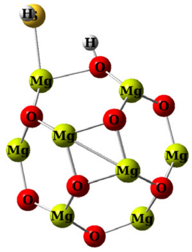 E_{ads} = -1.24 eV
2	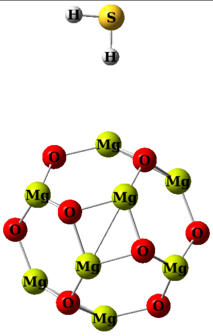	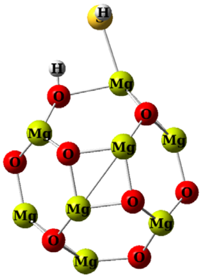	6	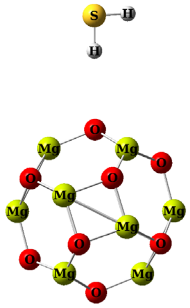	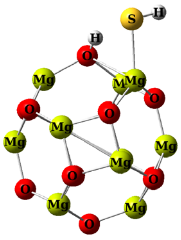

		Eads = -1.25 eV			Eads = -1.32 eV
3	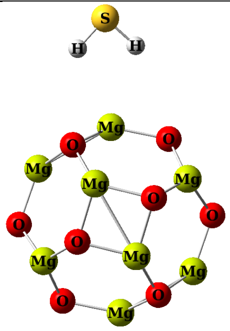	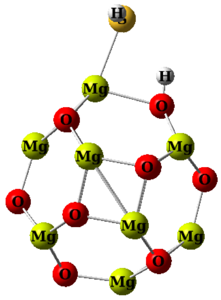	7	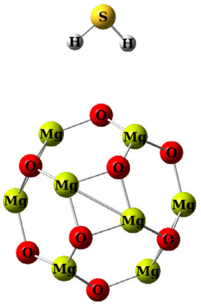	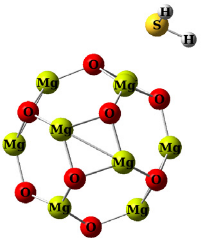
		Eads = -1.25 eV			Eads = -0.39 eV
4	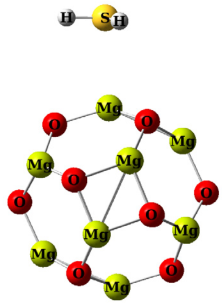	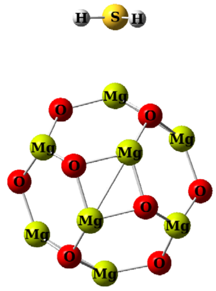	8	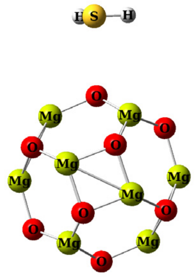	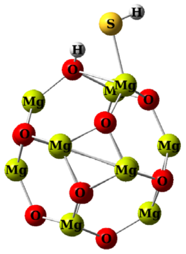
		Eads = -0.39 eV			Eads = -1.32 eV

Table S3. The examined orientations for SO_2 interaction with $Be_{12}O_{12}$.

mode	Input structure	Optimized structure	mode	Input structure	Optimized structure
1	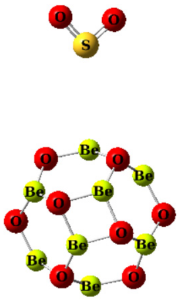	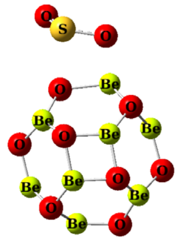	5	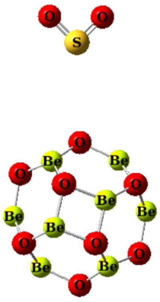	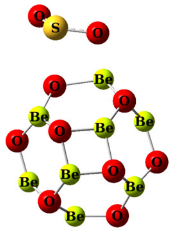
		Eads = -0.56 eV			Eads = -0.56 eV

2	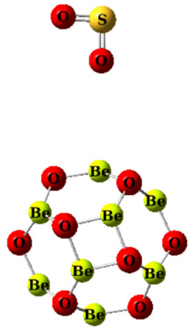	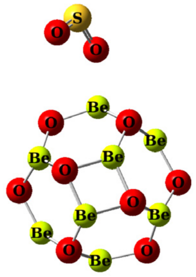	6	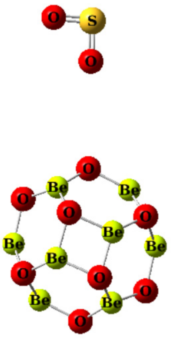	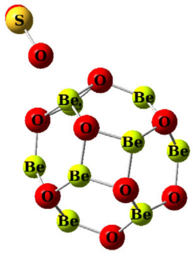
	E_{ads} = -0.51 eV			E_{ads} = -0.51 eV	
3	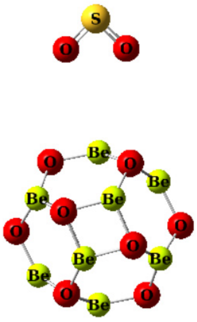	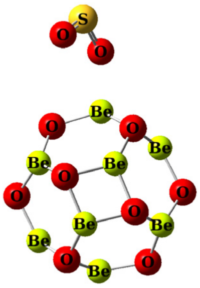	7	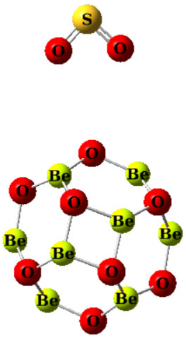	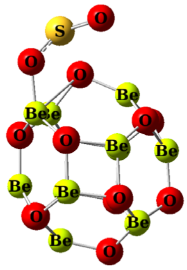
	E_{ads} = -0.51 eV			E_{ads} = -0.83 eV	
4	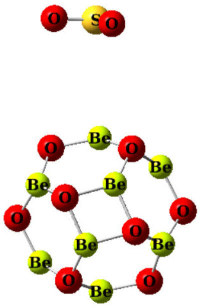	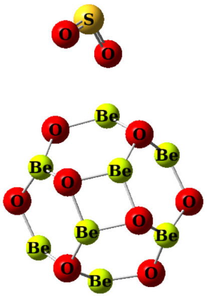	8	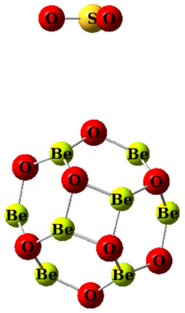	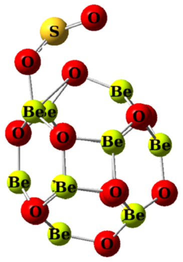
	E_{ads} = -0.51 eV			E_{ads} = -0.83 eV	

Table S4. The examined orientations for SO_2 interaction with $Mg_{12}O_{12}$.

mode	Input structure	Optimized structure	mode	Input structure	Optimized structure
1	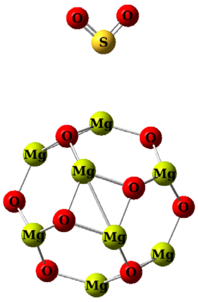	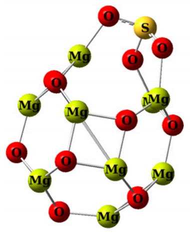	5	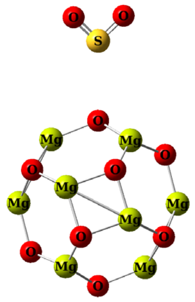	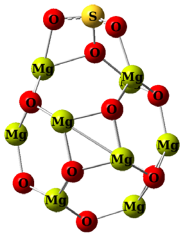
	E_{ads} = -2.33 eV			E_{ads} = -2.33 eV	

2	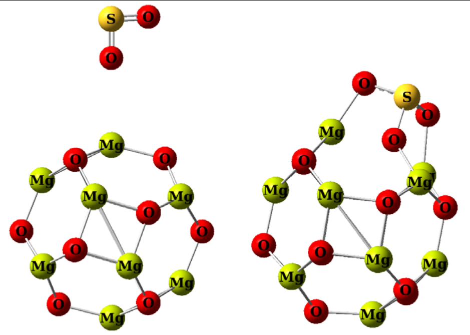<br>E_{ads} = -2.32 eV	6	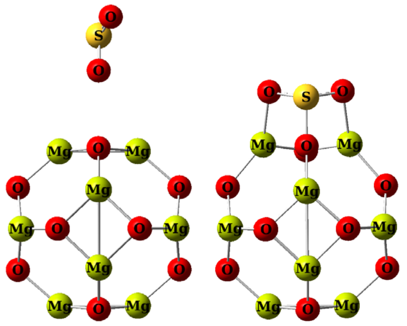<br>E_{ads} = -2.29 eV
3	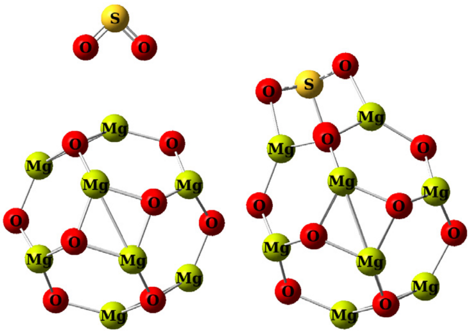<br>E_{ads} = -2.28 eV	7	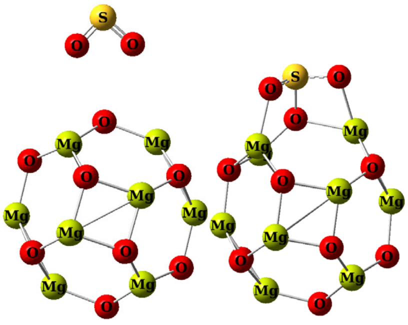<br>E_{ads} = -2.32 eV
4	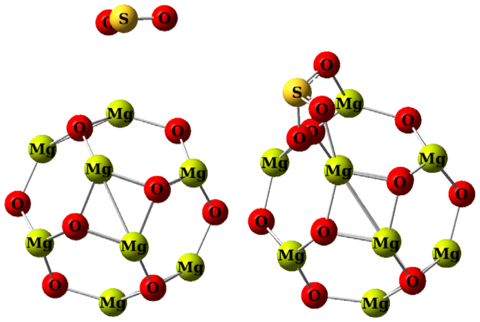<br>E_{ads} = -2.32 eV	8	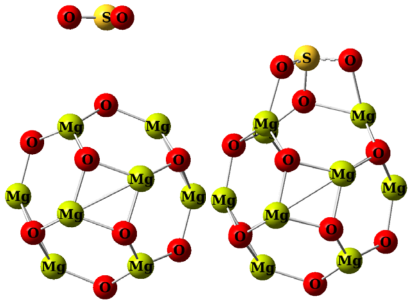<br>E_{ads} = -2.32 eV
